# Understanding the Role of Ureteral Access Sheath in Preventing Post-Operative Infectious Complications in Stone Patients Treated with Ureteroscopy and Ho:YAG Laser Lithotripsy: Results from a Tertiary Care Referral Center

**DOI:** 10.3390/jcm12041457

**Published:** 2023-02-12

**Authors:** Luca Villa, Pietro Dioni, Luigi Candela, Eugenio Ventimiglia, Mario De Angelis, Christian Corsini, Daniele Robesti, Margherita Fantin, Alessia D’Arma, Silvia Proietti, Guido Giusti, Ioannis Kartalas Goumas, Alberto Briganti, Francesco Montorsi, Andrea Salonia

**Affiliations:** 1 Division of Experimental Oncology/Unit of Urology, URI, IRCCS Ospedale San Raffaele, Via Olgettina, 20132 Milan, Italy; 2 School of Medicine, Vita-Salute San Raffaele University, 20132 Milan, Italy; 3 Unit of Urology, San Raffaele Turro, IRCCS Ospedale San Raffaele, 20132 Milan, Italy; 4 Department of Urology, Istituto Clinico Beato Matteo, 27029 Vigevano, Italy

**Keywords:** ureteroscopy, ureteral access sheath, fever, urosepsis, septic shock, urolithiasis

## Abstract

Introduction and objectives: The use of ureteral access sheaths (UAS) limits the irrigation-induced increase in intrarenal pressure during ureteroscopy (URS). We investigated the relationship between UAS and rates of postoperative infectious complications in stone patients treated with URS. Materials and methods: Data from 369 stone patients treated with URS from September 2016 to December 2021 at a single institution were analyzed. UAS (10/12 Fr) placement was attempted in case of intrarenal surgery. The chi-square test was used to assess the relationship between the use of UAS and fever, sepsis, and septic shock. Univariable and multivariable logistic regression analyses tested the association of patients’ characteristics and operative data and the rate of postoperative infectious complications. Results: Full data collection of 451 URS procedures was available. Overall, UAS was used in 220 (48.8%) procedures. As for postoperative infectious sequalae, we recorded fever (*n* = 52; 11.5%), sepsis (*n* = 10; 2.2%), and septic shock (*n* = 6; 1.3%). Of those, UAS was not used in 29 (55.8%), 7 (70%), and 5 (83.3%) cases, respectively (all *p* > 0.05). At multivariable logistic regression analysis, performing URS without UAS was not associated with the risk of having fever and sepsis, but it increased the risk of septic shock (OR = 14.6; 95% CI = 1.08–197.1). Moreover, age-adjusted CCI score (for fever-OR = 1.23; 95% CI = 1.07–1.42, sepsis-OR = 1.47; 95% CI = 1.09–1.99, and septic shock-OR = 1.61; 95% CI = 1.08–2.42, respectively), history of fever secondary to stones (for fever-OR = 2.23; 95% CI = 1.02–4.90) and preoperative positive urine culture (for sepsis-OR = 4.87; 95% CI = 1.12–21.25) did emerge as further associated risk factors. Conclusions: The use of UAS emerged to prevent the onset of septic shock in patients treated with URS, with no clear benefit in terms of fever and sepsis. Further studies may help clarify whether the reduction in fluid reabsorption load mediated by UAS is protective against life-threatening conditions in case of infectious complications. The patients’ baseline characteristics remain the main predictors of infectious sequelae in a clinical setting.

## 1. Introduction

Infectious complications following ureterorenoscopy and Ho:YAG lithotripsy (URS) in patients with urinary stones still represent a major clinical issue, with an overall incidence of 10% [1]. The irrigation-induced increase in intrarenal pressure during URS and the consequent pyelo-venous and pyelo-lymphatic backflow is thought to be the cause of bacterial translocation and bacteremia, and the trigger for developing clinical manifestations of infection with various degrees of severity [2,3]. Indeed, postoperative fever and urinary tract infections (UTI) may not only occur but also progress to urosepsis and septic shock, with potentially fatal consequences [4].

In this context, although various studies have demonstrated that the use of ureteral access sheaths (UAS) can reduce intrarenal pressure by promoting a constant outflow from the renal cavities [5,6], a direct benefit in terms of a reduction in perioperative infectious events has not been well established [7,8]. On the other hand, various clinical parameters resulted in a strong association with postoperative infectious complications in numerous series and may play a predominant role in promoting the occurrence of these harmful events [9,10,11,12,13].

Here, we aimed at clarifying the impact of UAS in terms of rates of infectious complications, by evaluating whether its use may impact the risk of postoperative infectious sequelae as stratified according to degrees of severity (i.e., fever, sepsis, and septic shock).

## 2. Methods

### 2.1. Study Population and Surgical Technique

Data from 369 consecutive patients who underwent ureteroscopy (URS) with intracorporeal Ho:YAG laser lithotripsy for ureteral and/or renal stones from September 2016 to December 2021 at a single institution were analyzed.

A preoperative urine culture was routinely obtained before each surgical procedure and any concomitant asymptomatic UTI was treated prior to surgery. Therefore, the procedures were scheduled at least 5 days after the beginning of a specific course of antibiotics according to the antibiogram findings. Moreover, antibiotic prophylaxis (specifically a second-generation cephalosporin) was administered preoperatively according to EAU Guidelines [14].

Patients were placed in the lithotomy position and general anesthesia was used to limit renal movement during breathing. All procedures were performed by two experienced endourologists (LV and EV) according to a standard technique. Specifically, a 0.035″ nitinol stiff guidewire (Orchestra from Coloplast, Humlebaek, Denmark) was used to engage the ureter. A semi-rigid 6/7.5 Fr ureteroscope (from Richard Wolf, Knittlingen, Germany) was used for stones located in the distal part of the ureter. A flexible ureteroscope (Flex-XC, Flex-X2 from Karl Storz, Germany; or URF-P5, URF-P6, URF-P7 from Olympus, Japan) was used for stones located above the iliac vessel. Ureteral access sheath (Re-Trace 10/12 Fr from Coloplast, Denmark) placement was attempted for stones located in the kidney or in the case of intrarenal retropulsion during the lithotripsy of ureteral stones. Ureteral access sheaths were not placed for ureteral stones lithotripsy. To achieve a constant pressure (40 cmH_2_O), active irrigation was provided by a 5 L saline bag placed 40 cm above the patient and connected to a manual pump (Traxerflow Dual Port from Rocamed, Monaco), which was activated when needed. A 100 W Ho:YAG laser (from Lumenis Inc., Yokne’am Illit, Israel) with a 200 µm laser fiber and a regular basket (ZeroTip 1.9 Fr from Boston Scientific, Marlborough, MA, USA) were used to perform the lithotripsy and to retrieve residual fragments, respectively. After all of the procedures, a DJ stent was placed and then removed in an outpatient setting within 7–10 days postoperatively.

Data collection followed the principles outlined in the Declaration of Helsinki. All patients signed an informed consent agreeing to share their own anonymous information for other future studies. The study was approved by the IRCCS San Raffaele Hospital Ethical Committee (Protocol Calcolosi—Endourologia, 19 September 2016).

### 2.2. Outcome Definition and Statistical Analysis

Postoperative fever was considered as any increase in body temperature above 38 °C.

Postoperative sepsis and septic shock were defined according to the Third International Consensus Definitions for Sepsis and Septic Shock [15].

Descriptive statistics were used to detail the clinical features of the whole cohort of patients.

The chi-square test was used to assess the relationship between the use of UAS and the occurrence of postoperative fever, sepsis, and septic shock.

Univariable and multivariable logistic regression analyses were performed to identify predictors of postoperative infectious complications. Covariates included patients’ characteristics: age-adjusted Charlson comorbidity index (CCI) score [16], history of fever secondary to stones, preoperative urine culture status, stone diameter, and operative data (i.e., presence of Double-J stent at surgery, operative time and the use of UAS).

All analyses were performed using SPSS v.24.0 (IBM Corp., Armonk, NY, USA). All tests were two-sided with a significance level set at *p* < 0.05.

## 3. Results

Out of 369 patients, we collected a complete set of data on 451 URS procedures, with 52 patients being treated with more than one surgery. Specifically, 317 (85.9%), 38 (10.3%), 10 (2.7%), and 4 (1.1%) patients underwent one, two, three, and ≥four URSs, respectively.

Detailed baseline patients’ characteristics are reported in Table 1. Overall, almost 70% of patients had no comorbidities, but one out of three (30.8%) had an age-adjusted CCI score ≥three. Overall, 12.5% and 10% of patients had a previous history of UTI and fever secondary to stones, respectively.

Perioperative and surgical data are reported in Table 2.

Out of 451 surgical procedures, fever, sepsis, and septic shock occurred in 52 (11.5%), 10 (2.2%), and 6 (1.3%) cases within 24 h after surgery, respectively. Out of 52 cases of fever, 41 (78.8%), 5 (9.6%), and 6 (11.6%) events occurred at the first, second and third URS or after, respectively. Out of 10 cases of urosepsis, 8 (80%) and 2 (20%) events occurred at the first and third URS, respectively. Out of 6 cases of septic shock, 5 (83.3%) and 1 (16.7%) events occurred at the first and third URS, respectively. Out of the patients who developed fever, sepsis, and septic shock, UAS was not used in 29 (55.8%), 7 (70%), and 5 (83.3%) cases, respectively (all *p* > 0.05).

Using multivariable logistic regression analysis to predict postoperative infectious complications, age-adjusted CCI score was associated with the risk of postoperative fever (*p* < 0.01; Table 3), sepsis (*p* = 0.01; Table 4), and septic shock (*p* = 0.02; Table 5), respectively. Likewise, history of fever secondary to stones was associated with the risk of postoperative fever (*p* = 0.04; Table 3) and sepsis (*p* = 0.04; Table 4). UAS insertion was associated with the risk of septic shock (*p* = 0.04; Table 5), but not with postoperative fever or sepsis.

## 4. Discussion

The development of a new energy source for lithotripsy has significantly contributed to the management of stone patients [17]. However, no major novelty has been recently introduced to improve the safety of stone patients treated with URS. Therefore, it is crucial to better understand the real impact of available tools to prevent or at least mitigate potential harmful conditions secondary to URS.

In our study, the rate of urosepsis following URS is 2.2%, which is slightly higher compared to the 0.1–0.5% prevalence reported by large observational international multicenter studies from the Clinical Research Office of the Endourological Society (CROES) [18,19]. Specifically, the CROES studies focused mainly on patients treated with URS for ureteral stones, whilst most patients in our study (i.e., two out of three) had stones in the intrarenal cavities (Table 2).

The potential harmful impact of an intrarenal procedure, as compared with a simple ureteroscopy, may be due to the increase in intrarenal pressure generated when the instrument is in the calicopyelic system. Indeed, Auge et al. demonstrated that the in vivo intrarenal pressure increased while advancing the ureteroscope toward the kidney compared to the distal ureter (59 vs. 51 cmH_2_O when the irrigation pressure was 200 cmH_2_O) [6]. Similarly, Jung and Osther registered very high pelvic pressures during retrograde intrarenal surgery, with pressure peaks up to 328 mm Hg (or 446 cmH_2_O) in the case of forced irrigation [2]. Therefore, having found the threshold for pyelo-venous backflow at 40–60 cmH_2_O [20], the natural consequences of operating with high intrarenal pressure may be renal extravasation and systemic fluid reabsorption.

Indeed, Loftus et al. have demonstrated in pig kidneys that the higher the intrarenal pressure of saline irrigation, the deeper the penetration of irrigation fluid into the renal parenchyma [21]. Therefore, this confirms the pathophysiology of potential bacterial transposition from the urine to the blood, along with bacteremia.

The intrarenal fluid reabsorption during URS has also been indirectly proved in humans by Cybulski et al. [22], by measuring fluid instilled into and collected from the urinary tract, with a mean estimated systemic fluid absorption during URS of 54 mL (range 4–137 mL).

The use of UAS has been demonstrated to promote a constant fluid backflow, limiting the irrigation-induced increase in intrarenal pressure and potentially the consequent pyelo-venous reflux, with a measured intrarenal pressure below the threshold for this phenomenon [5,6].

Current findings showed that 70% of urosepsis and 83.3% of septic shock events, respectively, occurred in those patients treated without the use of UAS. Conversely, no significant association was observed whether using UAS or not and the incidence of postoperative infectious complications at univariate analysis (Table 3, Table 4 and Table 5). The most probable reason for this observation comes from the low number of sepsis and septic shock events registered in our study (i.e., 10 (2.2%) and 6 (1.3%) cases, respectively). In this context, in a multicenter study from the CROES [7], Traxer et al. demonstrated that the use of UAS reduced the incidence of postoperative fever, UTI, and sepsis, although it was not clear whether the outcomes differed among the different centers involved. Moreover, although the irrigation flow rate emerged as an independent predictor of severe inflammatory response syndrome in a series of 260 stone patients treated with URS reported by Zhong et al. [8], the study lacked a control group of patients treated without UAS thus limiting the actual significance of their findings.

Therefore, demonstrating the real clinical impact of UAS in this setting is still a challenge.

In summary, the well-demonstrated increase in intrarenal pressure generated by an endoscopic procedure conducted without the use of UAS and the consequent more pronounced intrarenal liquid reabsorption do not automatically translate into a higher rate of postoperative infectious sequelae, which remain rare events, especially in their severe forms.

Moreover, a variety of other factors, including patient comorbidity, previous history of UTI, preoperative urine culture status, the presence of an indwelling DJ at the time of surgery—even with a negative preoperative urine culture—and long operative time may facilitate the development of these harmful infectious complications, as already demonstrated in a number of reports [9,13]. Here we confirm that age-adjusted CCI score emerged as the strongest predictor of all types of postoperative infectious complications.

The added value of the current study is that we found a significant benefit of UAS in preventing the risk of septic shock, after adjusting for other clinically relevant variables (Table 5). Therefore, we may comment on the current findings by hypothesizing that, despite the expected reduction in intrarenal reabsorption load during URS, the use of UAS is not able to completely prevent the risk of bacteremia and the consequent risk of postoperative infections, although it may be protective against a poor evolution toward life-threatening conditions.

The current study is not devoid of limitations. First, since we did not measure the intrarenal pressure during URS procedures, we can only speculate from the results of previous reports that the use of UAS could have reduced the forementioned parameter even in our cohort of patients, although we only had indirect proof through the intraoperative fluoroscopy showing an opacification of renal papillae along with the intrarenal collecting system after injection of medium contrast during URS without UAS, which conversely was not evident for URS cases with UAS. Secondly, we could not unequivocally demonstrate that the intrarenal reflux was the unique pathophysiological mechanism responsible for postoperative infectious complications, although it can be reasonably considered as the main trigger for these events in patients treated with URS. Thirdly, the numbers of postoperative sepsis and septic shock events were low in our series of consecutive standardized procedures. Therefore, larger studies are mandatory to help clarify whether the use of UAS is really protective against a poor evolution to life-threatening conditions rather than reducing any postoperative infectious complications.

## 5. Conclusions

Baseline characteristics of stone patients treated with URS emerged to be associated with the highest risk of postoperative infectious complications, whose rate is around 10%. The use of UAS emerged to prevent the onset of septic shock, with no clear benefit in terms of fever and sepsis outcomes. Further studies may help to clarify whether the UAS can mitigate the clinical impact of potential systemic bacterial dissemination caused by intrarenal fluid reabsorption during URS, rather than reducing the risk of any postoperative infectious complications.

## Figures and Tables

**Table 1:** Descriptive characteristics of the whole cohort of patients (*n* = 369). All values are shown as median (interquartile range) or frequency (proportion).

Variables	Overall = 369 Patients
Age at surgery (years)	
Median (IQR)	56 (46.4, 66)
Charlson Comorbidity Index (CCI) score, *n* (%)	
0	259 (70.2)
1	49 (13.3)
2	34 (9.2)
≥3	27 (7.3)
Age-adjusted CCI score, *n* (%)	
0	114 (30.8)
1	76 (20.6)
2	64 (17.3)
≥3	115 (31.1)
History of UTI, *n* (%)	
No	323 (7.5)
Yes	46 (12.5)
History of fever secondary to stones, *n* (%)	
No	332 (90)
Yes	37 (10)

Keys: Age-adjusted CCI score = CCI score + 0 if patient age is <50 years; CCI score + 1 if patient age ranges between 51 and 60 years; CCI score + 2 if patient age ranges between 61 and 70 years; CCI score + 3 if patient age ranges between 71 and 80 years; CCI score + 4 if patient age is >80 years. UTI = Urinary Tract Infection; IQR = Inter Quartile Range.

**Table 2:** Perioperative and surgical data of the 451 consecutive URS procedures. All values are shown as median (interquartile range) or frequency (proportion).

Variables	Overall = 451 Procedures
Pre-operative urine culture, *n* (%)	
Negative	379 (84)
Positive	72 (16)
Stone location, *n* (%)	
Ureter	144 (31.9)
Kidney	243 (53.9)
Ureter and Kidney	64 (13.2)
DJ stent placed before URS, *n* (%)	
No	227 (50.3)
Yes	224 (49.7)
Stone diameter (mm)	
Median (IQR)	11 (8.15)
Use of UAS, *n* (%)	
No	231 (51.2)
Yes	220 (48.8)
Operative time (min)	
Median (IQR)	63 (44, 89)
Postoperative fever, *n* (%)	
No	399 (88.5)
Yes	52 (11.5)
Postoperative sepsis, *n* (%)	
No	441 (97.8)
Yes	10 (2.2)
Postoperative septic shock, *n* (%)	
No	445 (98.7)
Yes	6 (1.3)

Keys: DJ = Double-J stent; UAS = Ureteral Access Sheath; IQR = Inter Quartile Range.

**Table 3:** Univariable and multivariable logistic regression analyses predicting postoperative fever.

Covariates	Univariate			Multivariate		
	OR	95% CI	*p*	OR	95% CI	*p*
Age-adjusted CCI score	1.29	1.13–1.49	<0.001	1.23	1.07–1.42	<0.01
History of fever secondary to lithiasis No vs. Yes	2.89	1.48–5.66	0.002	2.23	1.02–4.90	0.04
Preoperative urine culture Negative vs. Positive	2.41	1.25–4.70	0.009	1.56	0.72–3.35	0.26
DJ positioning at surgery No vs. Yes	1.32	0.74–2.36	0.35	0.94	0.49–1.78	0.84
Operative time (min)	1.01	1.00–1.02	0.14	1.01	0.99–1.01	0.09
Use of UAS Yes vs. No	0.81	0.45–1.45	0.48	1.64	0.88–3.07	0.12
Stone diameter (mm)	1.04	1.00–1.07	0.032	1.03	0.99–1.07	0.15

Keys: CCI = Charlson Comorbidity Index; DJ = Double-J stent; UAS = Ureteral Access Sheath.

**Table 4:** Univariable and multivariable logistic regression analyses predicting postoperative urosepsis.

Covariates	Univariate			Multivariate		
	OR	95% CI	*p*	OR	95% CI	*p*
Age-adjusted CCI score	1.51	1.16–1.98	<0.01	1.47	1.09–1.99	0.01
History of fever secondary to lithiasis No vs. Yes	6.47	1.82–23.04	<0.01	3.35	0.72–15.61	0.12
Preoperative urine culture Negative vs. Positive	8.47	2.33–30.85	<0.01	4.87	1.12–21.25	0.035
DJ positioning at surgery No vs. Yes	1.53	0.42–5.51	0.51	0.61	0.14–2.76	0.52
Operative time (min)	0.99	0.98–1.01	0.75	0.99	0.97–1.01	0.53
Use of UAS Yes vs. No	0.44	0.11–1.73	0.24	4.27	0.87–21.04	0.07
Stone diameter (mm)	1.03	0.96–1.10	0.41	1.05	0.96–1.16	0.30

Keys: CCI = Charlson Comorbidity Index; DJ = Double-J stent; UAS = Ureteral Access Sheath.

**Table 5:** Univariable and multivariable logistic regression analyses predicting postoperative septic shock.

Covariates	Univariate			Multivariate		
	OR	95% CI	*p*	OR	95% CI	*p*
Age-adjusted CCI score	1.56	1.11–2.19	0.01	1.61	1.08–2.42	0.02
History of fever secondary to lithiasis No vs. Yes	12.83	2.3–71.56	<0.01	7.64	0.94–62.01	0.057
Preoperative urine culture Negative vs. Positive	11.08	1.99–61.73	<0.01	5.84	0.72–47.57	0.09
DJ positioning at surgery No vs. Yes	2.04	0.37–11.28	0.41	0.59	0.07–5.01	0.63
Operative time (min)	0.99	0.96–1.02	0.52	0.98	0.95–1.02	0.36
Use of UAS Yes vs. No	0.21	0.02–1.78	0.15	14.6	1.08–197.1	0.04
Stone diameter (mm)	1.05	0.97–1.13	0.18	1.12	0.99–1.25	0.056

Keys: CCI = Charlson Comorbidity Index; DJ = Double-J stent; UAS = Ureteral Access Sheath.

## Data Availability

Data available on request due to restrictions (e.g., privacy). The data presented in this study are available on request from the corresponding author. The data are not publicly available due to patients’ privacy.
